# Inhibition of Fatty Acid Synthase Decreases Expression of Stemness Markers in Glioma Stem Cells

**DOI:** 10.1371/journal.pone.0147717

**Published:** 2016-01-25

**Authors:** Yuki Yasumoto, Hirofumi Miyazaki, Linda Koshy Vaidyan, Yoshiteru Kagawa, Majid Ebrahimi, Yui Yamamoto, Masaki Ogata, Yu Katsuyama, Hirokazu Sadahiro, Michiyasu Suzuki, Yuji Owada

**Affiliations:** 1 Department of Organ Anatomy, Yamaguchi University Graduate School of Medicine, Ube, Japan; 2 Department of Neurosurgery, Yamaguchi University Graduate School of Medicine, Ube, Japan; 3 Department of Organ Anatomy, Tohoku University Graduate School of Medicine, Sendai, Japan; Wayne State University, UNITED STATES

## Abstract

Cellular metabolic changes, especially to lipid metabolism, have recently been recognized as a hallmark of various cancer cells. However, little is known about the significance of cellular lipid metabolism in the regulation of biological activity of glioma stem cells (GSCs). In this study, we examined the expression and role of fatty acid synthase (FASN), a key lipogenic enzyme, in GSCs. In the *de novo* lipid synthesis assay, GSCs exhibited higher lipogenesis than differentiated non-GSCs. Western blot and immunocytochemical analyses revealed that FASN is strongly expressed in multiple lines of patient-derived GSCs (G144 and Y10), but its expression was markedly reduced upon differentiation. When GSCs were treated with 20 μM cerulenin, a pharmacological inhibitor of FASN, their proliferation and migration were significantly suppressed and *de novo* lipogenesis decreased. Furthermore, following cerulenin treatment, expression of the GSC markers nestin, Sox2 and fatty acid binding protein (FABP7), markers of GCSs, decreased while that of glial fibrillary acidic protein (GFAP) expression increased. Taken together, our results indicate that FASN plays a pivotal role in the maintenance of GSC stemness, and FASN-mediated *de novo* lipid biosynthesis is closely associated with tumor growth and invasion in glioblastoma.

## Introduction

Of the gliomas, glioblastoma has the poorest prognosis, with a median survival rate between 1 and 2 years [1]. Glioblastoma is resistant to radiation and chemotherapy, and glioma stem cells (GSCs) are thought to be partially responsible for the resistance to therapy, recurrence, and invasiveness [2, 3]. Therefore, elucidating the molecular mechanisms that regulated the biological activity of GSCs is important for identifying novel therapeutic targets.

Metabolic reprogramming is thought to be a new hallmark of cancer [4]. Cancer cells including glioblastoma has been reported to rely on glycolysis than on oxidative phosphorylation for their energy metabolism, which is known as the Warburg effect [5]. Another metabolic feature of cancer cells is deregulated fatty acid and cholesterol biosynthesis, especially *de novo* lipogenesis [6]. Monoacylglycerol lipase (MAGL) a key enzyme for *de novo* fatty acid synthesis pathway, is recently shown to be upregulated in malignant tumors and facilitate high levels of cellular β-oxidation [7]. In glioma, it is recently reported that fatty acid binding protein (FABP7), which is one of the intracellular lipid chaperones mediating the cellular dynamics of fatty acids, is highly expressed in glioblastoma neurospheres [8], and that manipulation of FABP7 level in malignant glioma cells affects their proliferation and migration features [9, 10]. However, the precise mechanism and significance of fatty acid metabolism in the glioma cells remains largely unknown.

Fatty acid synthase (FASN) is an intracellular enzyme capable of *de novo* lipogenesis by catalyzing the conversion of acetyl-CoA and malonyl-CoA to palmitate [11]. FASN expression is drastically increased under pathological conditions [6], and it has been associated with the development of a variety of diseases including cardiovascular disease [12], insulin resistance of type 2 diabetes [13] and many types of cancers [6]. FASN overexpression has been reported in several cancers, including glioma [14], breast [15], prostate [16], colon [17], and lung [18] cancer, compared with their respective normal tissue. Recently, FASN expression was shown to be notably upregulated in inducible pluripotent stem (iPS) cells, and pharmacological inhibition of FASN activity markedly decreased the reprogramming efficiency of iPS cells [19]. This finding suggested that *de novo* fatty acid synthesis mediated by FASN is important for maintaining stemness. FASN was also found to be highly active in adult neural stem and progenitor cells (NSPCs), and conditional deletion of FASN in mouse NSPCs impaired adult neurogenesis [20]. However, the contribution of FASN to the maintenance of cancer stem cells remains unclear.

In the present study, we determined the role of FASN in GSCs. We observed that *de novo* fatty acid synthesis is highly active and FASN expression is upregulated in GCS. We also found that pharmacological inhibition of FASN dramatically inhibits cell proliferation and invasiveness of GSCs, and alters the expression of several stem cell markers. Collectively, our data suggest that *de novo* fatty acid synthesis mediated by FASN is essential for regulation of the biological features of GSCs.

## Materials and Methods

### Tumor samples and development of patient-derived GSC lines

Glioma tissue samples for histopathological examination were derived from patients, who underwent tumor resection after glial tumor diagnosis at the Yamaguchi University Medical Center. Pathological specimens used in the immunohistochemical study were derived from a 70-year-old male patient. The study was approved by the Ethics Committee of Yamaguchi University (No. H21-49-3). The study protocol followed the principles outlined in the Declaration of Helsinki. Written informed consent was obtained from all patients.

The GSC lines, G144 and G179, were kindly gifted by Dr. Peter Dirks (Arthur and Sonia Labatt Brain Tumor Research Center, Toronto, Canada) [21]. The GSC lines Y10, Y02, Y04, Y14 was generated by us as described before [22], using surgically resected tumors from an 80-year-old male, 70-year-old woman, 63-year-old male, 71-year-old male patient diagnosed with glioblastoma multiforme. Tumors were dissociated into single cells using trypsin (1.33 mg/mL), hyaluronidase (0.67 mg/mL), and kynurenic acid (0.17 mg/mL) for 50 min at 37°C. The dissociated cells were cultured in serum-free neurobasal medium supplemented with EGF (20 ng/mL) and FGF (20 ng/mL) to form neurospheres in suspension culture. The neurospheres were subsequently dissociated into single cells by placing in Accutase, and then the cells were transferred to fresh culture vessels coated with poly-L-ornithine and 1% Laminin overnight at 37°C. GSCs were expanded using serum-free media supplemented with B27, hormone mix, EGF (20 ng/mL), and FGF (20 ng/mL). After reaching confluency, GSCs were dissociated using Accutase to give a single cell solution and were split 1:5 to 1:8 for passage culture. The medium was replaced every 3–4 days. Each GSC line was differentiated into a non-GSC line in DMEM supplemented with 10% fetal bovine serum (FBS), without EGF or FGF for 9 days or 21 days. Neurobasal medium and B27 were purchased from Invitrogen (Carlsbad, CA, USA). FBS was purchased from HyClone Laboratories, Inc. (Logan, UT, USA). All other chemicals and reagents were purchased from Sigma-Aldrich Corporation (St. Louis, MO, USA).

### *De novo* lipid synthesis assay

*De novo* lipid synthesis was analyzed using [^14^C]-glucose (PerkinElmer, Boston, MA, USA) and [^14^C]-acetate (PerkinElmer). Cells were cultured to confluence. The medium was replaced with a fresh one without FBS just before adding the radioisotopes. Cells were exposed to [^14^C]-glucose for 24 h and [^14^C]-acetate for 8 h without antibiotics. The cells were then washed in PBS and lysed using scrapers. Protein content was measured by the bicinchoninic acid method (Pierce, Rockfold, IL, USA). Total lipids were extracted by the Bligh-dyer method [23]. Briefly, 0.5 ml of methanol and 0.25 ml of chloroform was added. Tubes were shaken vigorously for 2 min, and then incubated for more than 20 min at room temperature. Next, 0.2 ml of chloroform and 0.2 ml of PBS were added and vortexed, and then the mixuture was centrifuged at 3000 rpm for 20 min. The chloroform layer was collected using a Pastur pipette. After evaporation at 35°C under the N2 gas, lipids were dissolved with hexane/methanol (19:1). Subsequently, 5 ml of Ultima gold liquid scintillation counting cocktail (PerkinElmer) was added to each vial and the radioactive was detected using the Aloka radioisotope counter machine. The results were calculated as DPM/μg protein.

### RNA preparation and quantitative Real-Time PCR

Total RNA was isolated with TRIzol (Invitrogen) according to the manufacturer’s instructions, treated with DNase (Promega Corporation, Madison, WI, USA), and re-extracted using phenol/chloroform and LiCl precipitation. RNA concentration was determined using NANODROP LITE (Thermo Scientific, Hudson, NH, USA). First-strand cDNA synthesis was performed with Transcriptor High Fidelity reverse transcriptase kit (Roche Diagnostics, GmbH, Mannheim Germany) using oligo d(T) primers. Quantitative Real-Time PCR (qPCR) was performed using the Taqman^®^ Universal PCR Master Mix kit (Applied Biosystems, Carlsbad, CA, USA) and Taqman Gene Expression Assays for nestin (Hs04187831_g1), CD133 (prominin1) (Hs01009250_m1), Sox2 (Hs00415716_m1), FABP7 (Hs00361426_m1) and 18SmRNA (Hs03928985_g1). Reactions were performed in triplicate using 96-well optical plates on a StepOnePlus Real-Time PCR System (Applied Biosystems).

### Transplantation of GSCs into NOD-SCID mouse brain

All experimental procedures involving mice were approved by the Institute of Laboratory Animals of Yamaguchi University. Adult male and female NOD-SCID mice weighing 20–25 g (5 weeks old) were used in the study. Surgical procedures were performed under sterile condition. Mice were anesthetized by intraperitoneal injection of ketamine and xylazine, and then positioned in a rodent stereotaxic frame. Subsequently 100,000 GSCs in 2 μl of cold PBS were stereotactically injected into the right putamen (1 mm forward, 2 mm right lateral from the bregma, and 2.5 mm down from the dura) using a Hamilton syringe and a 30 G needle. The inoculation period was 2 min and the needle was left in place for another 5 min before withdrawal. Mice were returned to their cages and monitored for signs of illness. Following transplantation, animal health was monitored every day for up to 22 weeks. Mice were euthanized if they were unable to eat or drink. Mice were sacrificed at approximately 22 weeks after transplantation. Brains were used for preparation of histological sections. Primary antibody staining using anti-FASN (Sigma-Aldrich, 1:100) was carried out after deparaffinization, followed by incubation with biotinylated anti-rabbit IgG (Vector Laboratories, Burlingame, CA, USA, 1:200). The immunoreaction sites were visualized using the avidin-biotinylated peroxidase complex (ABC) system (Vector Laboratories) using diaminobenzidine as a substrate.

### Immunostaining

Double immunostaining of FASN and Sox2 was performed following the protocol as described previously [24]. Briefly, tumor samples were fixed with 10% formalin in 0.1 M phosphate buffer (pH 7.4) and were then dehydrated and embedded in paraffin. Paraffin sections were deparaffinized, washed thoroughly with water, and treated with microwave in HISTOFINE (pH 9) (Nichirei, Tokyo, Japan) for 40 min. After washing with TPBS for 5 min, sections were blocked in Protein Block Serum-Free Ready-To-Use (Dako Japan, Tokyo, Japan) for 10 min and incubated with rabbit anti-FASN antibody (Sigma-Aldrich, 1:100) for 60 min, followed by incubation with alkaline phosphatase-conjugated donkey anti-rabbit IgG (Jackson ImmunoResearch Laboratories, West Grove, PA, USA, 1:50) for 30 min. The chromogenic reaction was performed by using a Vulcan Fast Red Chromogen Kit 2 (BIOCARE MEDICAL, Concord, CA, USA) according to the manufacture’s manual. After using denaturing solution (BIOCARE MEDICAL) to denature the alkaline phosphatase, sections were incubated with goat anti-Sox2 antibody (Santa Cruz Biotechnology, Dallas, TX, USA, 1:100) and subsequently with alkaline phosphatase-conjugated donkey anti-goat IgG (Jackson ImmunoResearch Laboratories, 1:50). Subsequently, the chromogenic reaction was performed using PermaBlue/AP (Diagnostic BioSystems, Pleasanton, CA, USA) according to the manufacture’s manual. The sections were air-dried and cover slipped.

For immunocytochemical analysis, cells were fixed in 4% paraformaldehyde for 15 min. After blocking in 5% goat serum for 40 min, the cells were incubated overnight at 4°C with combinations of the following primary antibodies: rabbit anti-FASN (1:100), mouse anti-nestin (Millipore Corporation Billerica, MA, USA, 1:200), goat anti-Sox2 (1:500) antibody, or mouse anti-CD133 (Miltenyi Biotec Inc., Auburn, CA, USA, 1:50). The cells were incubated for 30 min at RT with suitable combinations of the following secondary antibodies: Alexa Fluor^®^ 488 goat anti-rabbit IgG, Alexa Fluor^®^ 488 goat anti-mouse IgG, Alexa Fluor^®^ 568 goat anti-rabbit IgG, or Alexa Fluor^®^ 568 donkey anti-goat IgG (Invitrogen, 1:500 for all). 4', 6-Diamidino-2-phenylindole (DAPI) (Invitrogen, 0.5 μg/ml,) was added as a nuclear marker. Cells were cover-slipped using Fluoromount (Diagnostic BioSystems). Images were acquired using a confocal laser scanning microscope (LSM510 META; Carl Zeiss, Oberkochen, Germany).

### Western blotting

Cells or tissues were lysed using buffer containing 100 mM Tris-Cl (pH 6.8), 4% SDS, 20% glycerol, and 200 mM β-mercaptoethanol. Protein was quantified by the BCA Protein assay (Pierce) using a Viento multi-spectrophotometer at 562 nm. Equal amounts of protein (15–30 μg) were separated by SDS-PAGE, transferred to Immobilon PVDF membranes (Millipore), and blocked with either 5% skimmed milk or rabbit serum in TBST (10 mM Tris (pH 7.5), 100 mM NaCl, and 0.1% Tween 20). Membranes were incubated overnight at 4°C with primary antibodies diluted in blocking buffer. The following primary antibodies were used: anti-FASN (Sigma-Aldrich, 1:1000), anti-CD133 (Miltenyi Biotec, 1:100), anti-FABP7 (Owada et al., 2006, 1:1000), anti-nestin (Millipore, 1:5000), anti-Sox2 (Millipore-Chemicon 1:1000), and anti- β-actin (Santa Cruz Biotechnology, 1:5000) antibody. Blots were washed and incubated with horseradish peroxidase-conjugated secondary antibodies for 1 h at RT. Immunoreactive protein bands were visualized using ECL western blotting detection reagents (GE Healthcare UK Ltd, Amersham Place, England).

### Cell viability

To evaluate cell viability, MTS [3-(4,5-dimethylthiazol-2-yl)-5-(3-carboxymethoxyphenyl)-2-(4-sulfophenyl)-2H-tetrazolium, inner salt] assay was performed using CellTiter 96 Aqueous nonradioactive cell proliferation assay kit (Promega, Madison, WI, USA) following the manufacturer’s instructions. Briefly, cells were plated into 96-well plates at 8,000 cells/well with indicated concentrations of cerulenin for the indicated times. After 0, 24, 48, 72h, MTS solution was added to the plate. After 2.5 h, the absorbance at 490 nm was measured using the ARVO^™^ X Multilabel Plate Reader (PerkinElmer).

### Migration assay

The invasiveness of GSCs was measured using Matrigel invasion assay. Briefly, Transwell inserts (Corning Life Sciences, Corning, NY, USA) with 8 μm pore size were coated with 200 μg/ml Matrigel (BD Bioscience, San Jose, CA, USA). In addition to FGF and EGF, the upper wells contained DMEM/F-12 medium without B27 supplement and hormone mix, or DMEM/F-12 supplemented with B27 and hormone mix as an attractant for the lower wells. GSCs were plated at 20,000 cells/well in duplicate. After 48 h of incubation, the noninvading cells in the upper chambers were gently wiped away, and the adherent cells on the lower side of each membrane were fixed and stained with 1% toluidine blue solution. Five fields were randomly selected and counted under a microscope. Invasion was quantified by counting stained cells through the coated membranes, and the mean count was calculated for statistical analysis.

### Sphere forming assay

Sphere forming assay was performed following the protocol by Singh et al., with slight modifications [25]. GSCs (Y02, Y04 and Y10 cells) were suspended and plated onto non-adherent 96-well flat-bottom plates at 8,000 cells/well in 0.2 ml of GSC medium. The cells were incubated at 37°C for 7 days and then the number and the maximum diameter of spheres were determined.

### Statistical analysis

All numerical data are shown as means ± SEM. For qPCR and western blotting results, the experimental groups were compared using unpaired Student’s t-test. P < 0.05 was considered significant.

## Results

### *De novo* lipogenesis was high in GSCs

**Fig 1:**
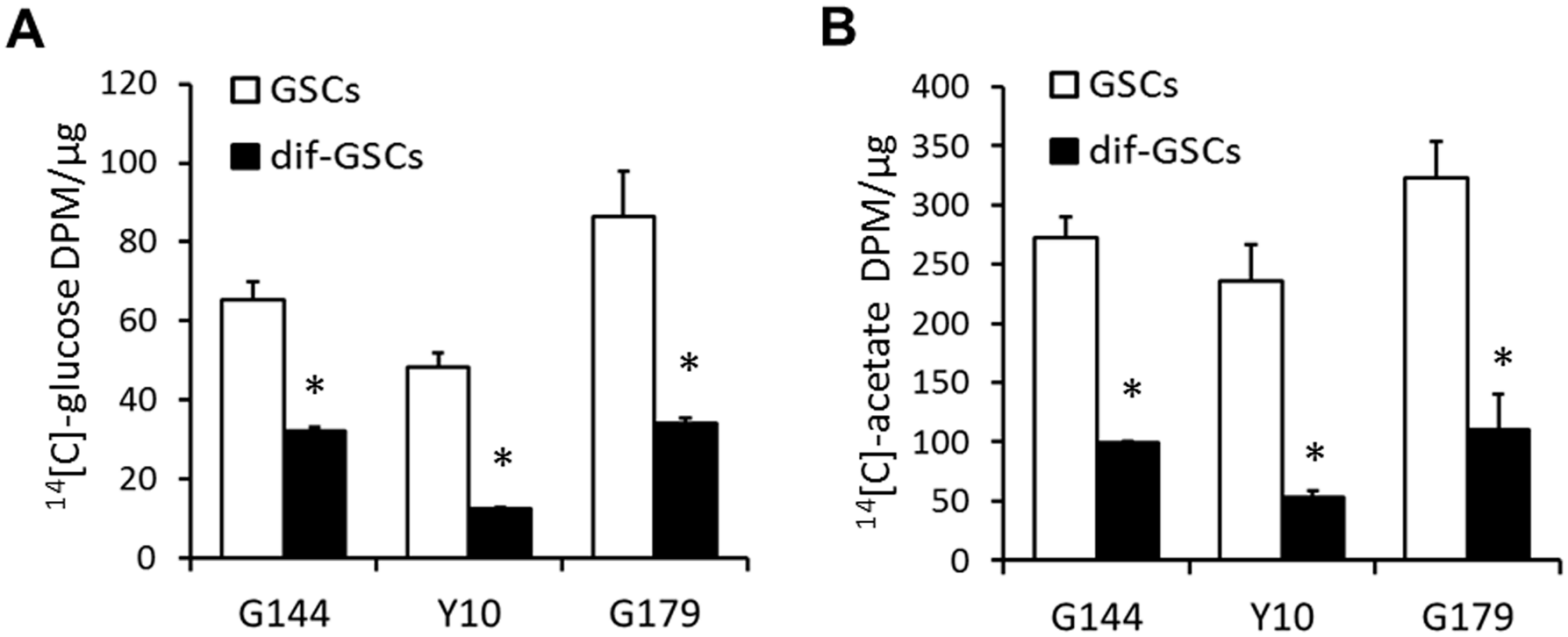
*De novo* lipogenesis using glucose and acetate as carbon source in GSCs and differentiated non-GSCs. G144, Y10 and G179 cells were incubated with [^14^C]-glucose (A) or [^14^C]-acetate (B) for 24 or 8 h to measure glucose or acetate incorporation into total lipid, respectively. Data are represented as means ± SEM for three independent experiments for each cell line. * P < 0.01.

We first explored whether incorporation of glucose or acetate into the lipid fraction representing *de novo* lipogenesis [26] is altered between GSCs and differentiated non-GSCs. ^14^[C]-Glucose incorporation was significantly higher in GSCs than in differentiated non-GSCs (65.38 ± 4.45 DPM/μg in G144; 48.16 ± 3.76 DPM/μg in Y10; 86.52 ± 11.30 DPM/μg in G179 vs. 32.32 ± 0.82 DPM/μg in dif-G144; 12.38 ± 0.48 DPM/μg in dif-Y10; 34.09 ± 1.38 DPM/μg in dif-G179; P < 0.01; Fig 1A). Similar results were obtained for the incorporation of ^14^[C]-acetate into lipids (271.73 ± 18.26 DPM/μg in G144; 235.82 ± 30.84 DPM/μg in Y10; 323.07 ± 29.91 DPM/μg in G179 vs. 99.62 ± 0.98 DPM/μg in dif-G144; 52.86 ± 5.33 DPM/μg in dif-Y10; 110.08 ± 29.65 DPM/μg in dif-G179, P < 0.01; Fig 1B). These results suggest that *de novo* lipogenesis was higher in GSCs than in differentiated non-GSCs.

### FASN expression was high in patient-derived GSCs

**Fig 2:**
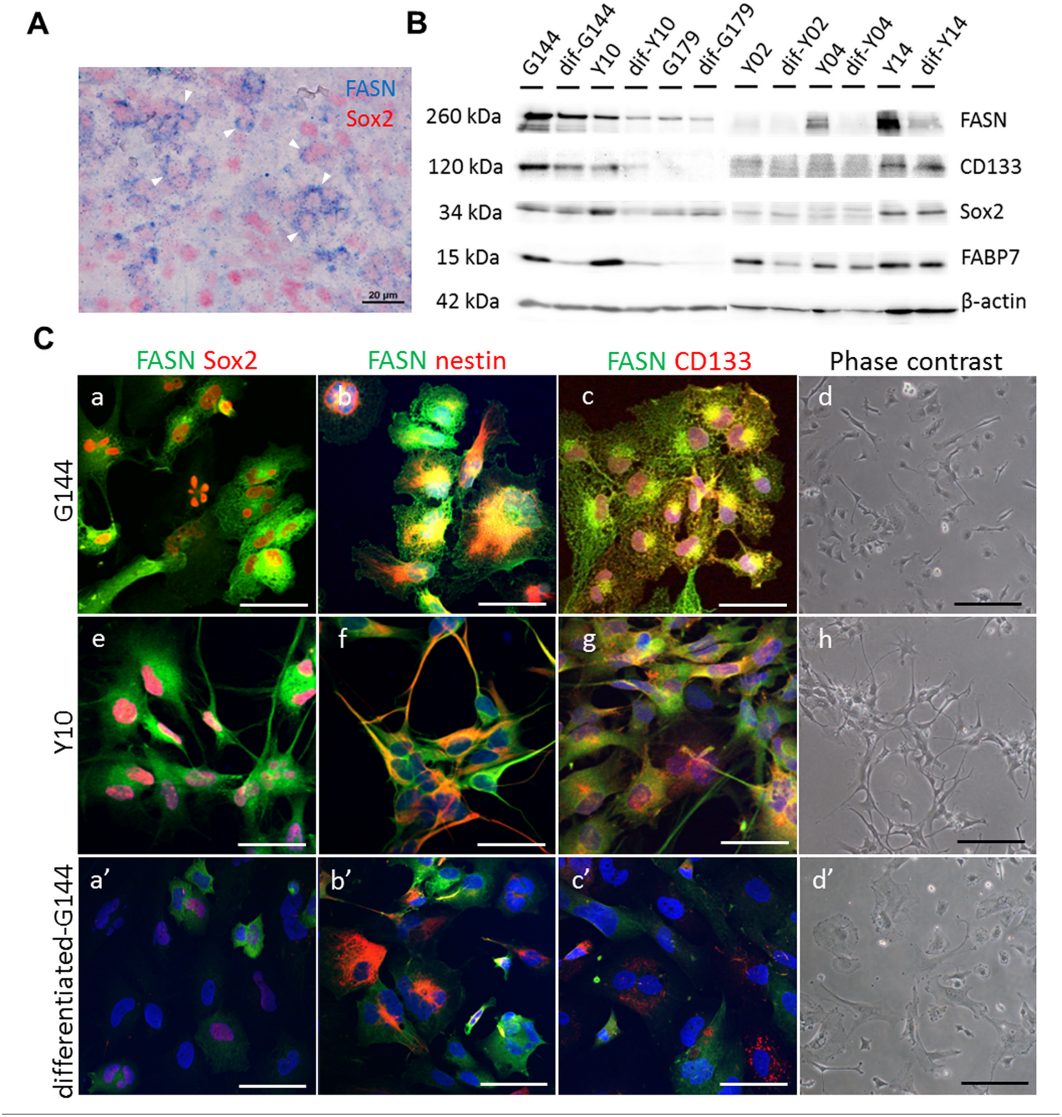
Expression of FASN in human glioblastoma cells and GSC lines. (A) Human glioblastoma cells show the strong expression of FASN (blue) and the neural stem cell marker, Sox2 (red). Sox2 expression is confined to the nuclei, whereas FASN is expressed in the cytosol. The white arrows show FASN^+^Sox2^+^ cells. (B) Western blotting showing the expression of FASN, CD133, Sox2, and FABP7 in G144, Y10, G179, Y02, Y04 and Y14 GSC lines. Upon differentiation in the presence of FBS, FASN expression, similar to that of CD133, Sox2, and FABP7, was down-regulated. Expression of β-actin was used as an internal control. (C) Expression of FASN in GSC lines before and after differentiation. GSCs show strong expression of FASN and other neural stem cell markers, Sox2, nestin and CD133. Upon differentiation in the presence of FBS, FASN expression is down-regulated, similar to that of Sox2, nestin, and CD133. Immunofluorescence micrographs showing the co-expression of FASN with Sox2 (a, e, a’) and nestin (b, f, b’) in G144 and Y10 GSC lines. Phase contrast micrographs showing the morphology of GSC lines (G144 and Y10) in the presence of EGF and FGF (d, h) or after differentiation in the presence of FBS (d’). Bars in a-c, e-g, a’-c’ = 50 μm, Bars in d, h, d’ = 20 μm.

In this study, FASN was highly detected in the cytoplasm of human glioblastoma cells, and FASN was frequently co-localized with Sox2, a marker of GSC (Fig 2A). FASN immunoreactivities were detected in the cytosol in tumor cells, while those of Sox2 were confined to the nuclei (Fig 2A). Therefore, we next investigated the expression of FASN in GSCs by qPCR and western blot analysis. qPCR analysis revealed that FASN mRNA levels in GCSs decreased after differentiation (S1 Fig). In western blot, FASN levels in GSCs were markedly decreased after induction of differentiation (Fig 2B). Of note, such decrease in FASN expression during GSC differentiation is well correlated with changes in the expression of CD133, Sox2 and FABP7 (Fig 2B, S1 Fig), all of which have been shown to regulate the biology of GSCs [8, 24, 27, 28]. These data suggest that FASN might be involved in the maintenance of GSC stemness, possibly through the regulation of cellular fatty acid homeostasis. In immunohistochemistry, strong FASN expression was detected in the cytoplasm of G144 and Y10 GSCs, and it was found to be co-expressed with stem cell markers, such as Sox2, nestin, and CD133 (Fig 2Ca–2Ch). Interestingly, FASN expression was markedly decreased in differentiated G144, in which the expression of stem cell markers was also decreased (Fig 2Ca’–2Cd’). In addition, we found high levels of FASN in transplanted GSCs in a xenograft mouse model (S2 Fig).

### FASN inhibition decreased invasiveness of GSCs

**Fig 3:**
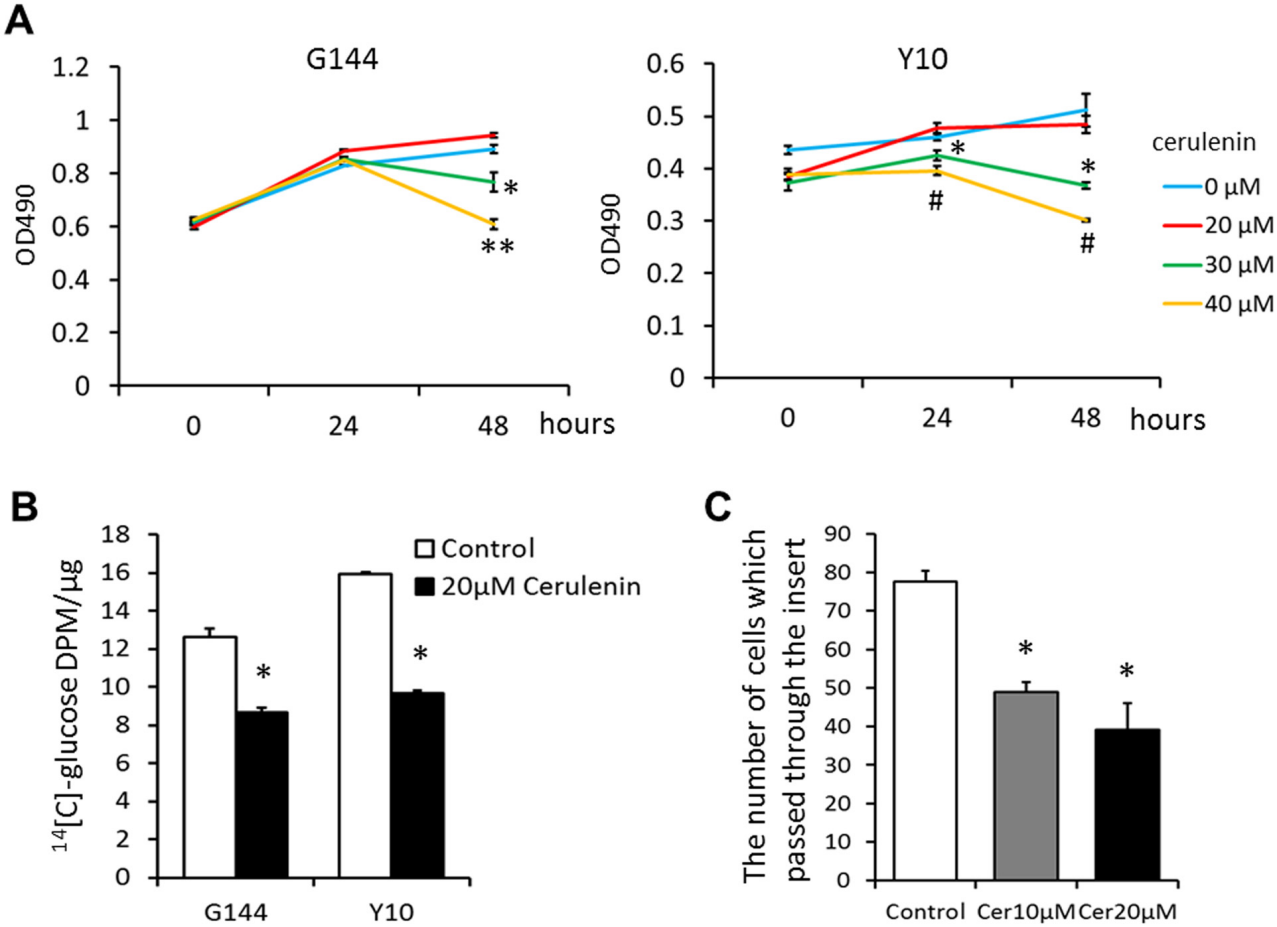
Effects of cerulenin on GSC viability and invasion. (A) Effects of cerulenin on GSC viability. Cell viability was assessed 0, 24, 48 h treatment with 0, 20, 30, 40 μM cerulenin by MTS assay. G144 and Y10 cell viability was significantly reduced by 30, 40 μM cerulenin. * P < 0.05, ^#^ P < 0.01, ** P< 0.001 (B) *De novo* lipogenesis using glucose as carbon source in GSCs and GSCs incubated with 20 μM cerulenin. * P < 0.05 (C) Quantitative results of Matrigel invasion assay. Each column represents mean ± SEM for three independent experiments. * P < 0.05.

Cerulenin, a specific inhibitor of FASN, binds to the active site of the condensing region of FASN, thereby inactivating a key step in fatty acid synthesis [29]. We first examined the effect of cerulenin on the proliferation of GSCs (G144 and Y10), and found that 30 or 40 μM cerulenin significantly decreased the number of surviving GSCs, while 20 μM showed no significant effect (Fig 3A). We also noted that 30 μM cerulenin treatments significantly affected the viability of GSCs, while it had no effect on glioblastoma cell lines (U373 and U87) (S3 Fig). GSCs did not show any marked changes in their morphology at 48h after 20 μM cerulenin treatment (data not shown), but *de novo* lipogenesis was significantly decreased (by approximately 40%) (Fig 3B). Furthermore, in the Matrigel invasion assay, the number of GSCs passed through the gel was significantly decreased by 40% or 50% when GSCs were treated with 10 μM or 20 μM of cerulenin, respectively (Fig 3C). Collectively, these results show that extensive FASN inhibition in GSCs affects their survival and that *de novo* lipogenesis in GSCs is essential of invasiveness, a unique characteristic of GSCs [25, 30].

### FASN inhibition decreased sphere formation of GSCs

**Fig 4:**
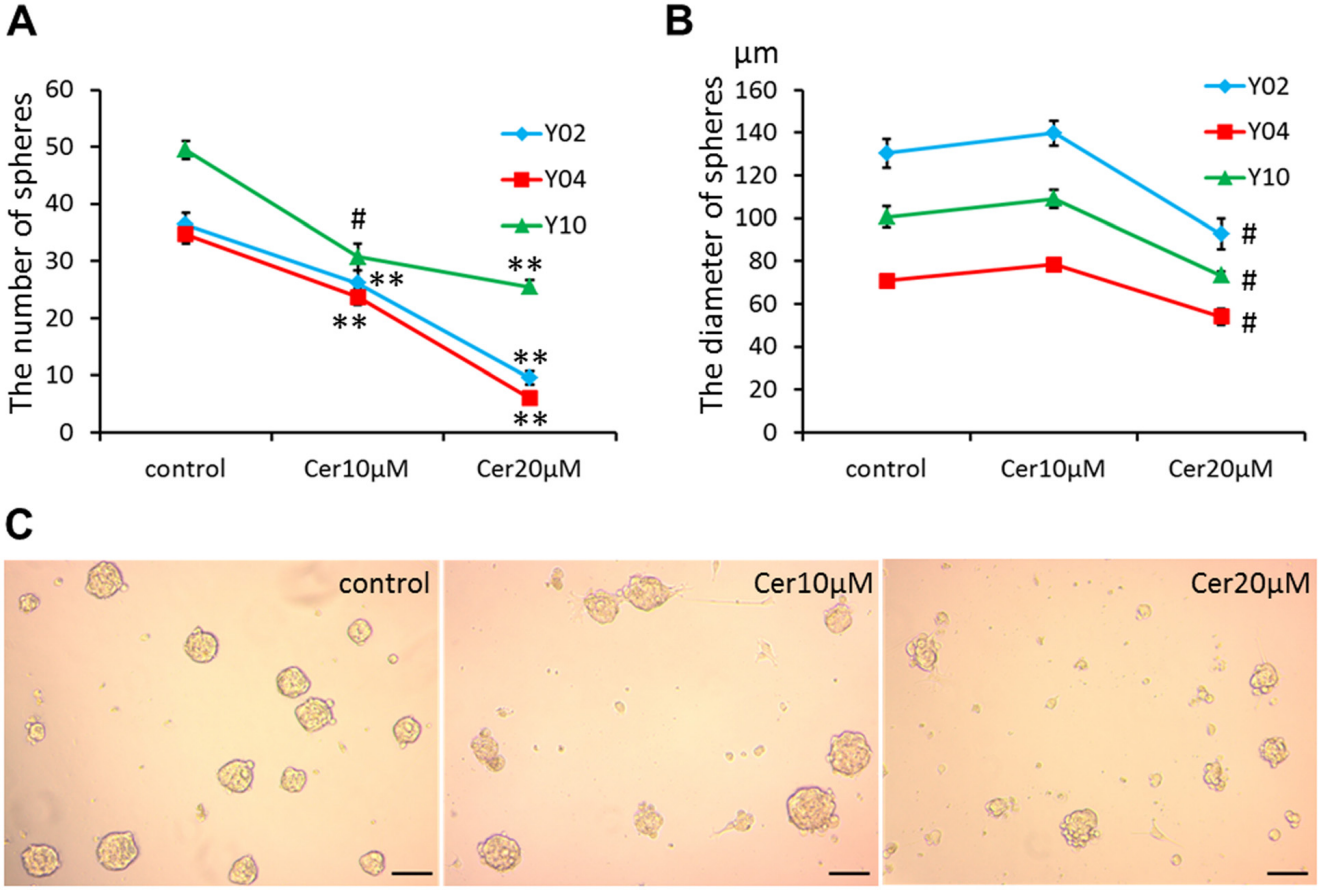
Effects of cerulenin on sphere formation. The number of spheres (A) and the diameter of GSC sphere (B) treated with 0 (control), 10, 20 μM cerulenin. ^#^ P < 0.01, ** P< 0.001 compared with control. (C) Representative phase contrast micrographs showing the morphology of Y10 in the control and the presence of 10 and 20 μM cerulenin. Bars = 100 μm.

We examined the effect of cerulenin on the sphere formation of GSCs (Y02, Y04 and Y10), and found that 10 or 20 μM cerulenin significantly decreased the number of spheres of GSCs (Fig 4A). GSCs did not show any marked changes in their morphology after 10 μM cerulenin treatment, but the diameter of GSC spheres was significantly reduced after 20 μM cerulenin treatment (Fig 4B and 4C).

### Inhibition of FASN decreased expression of stemness markers

**Fig 5:**
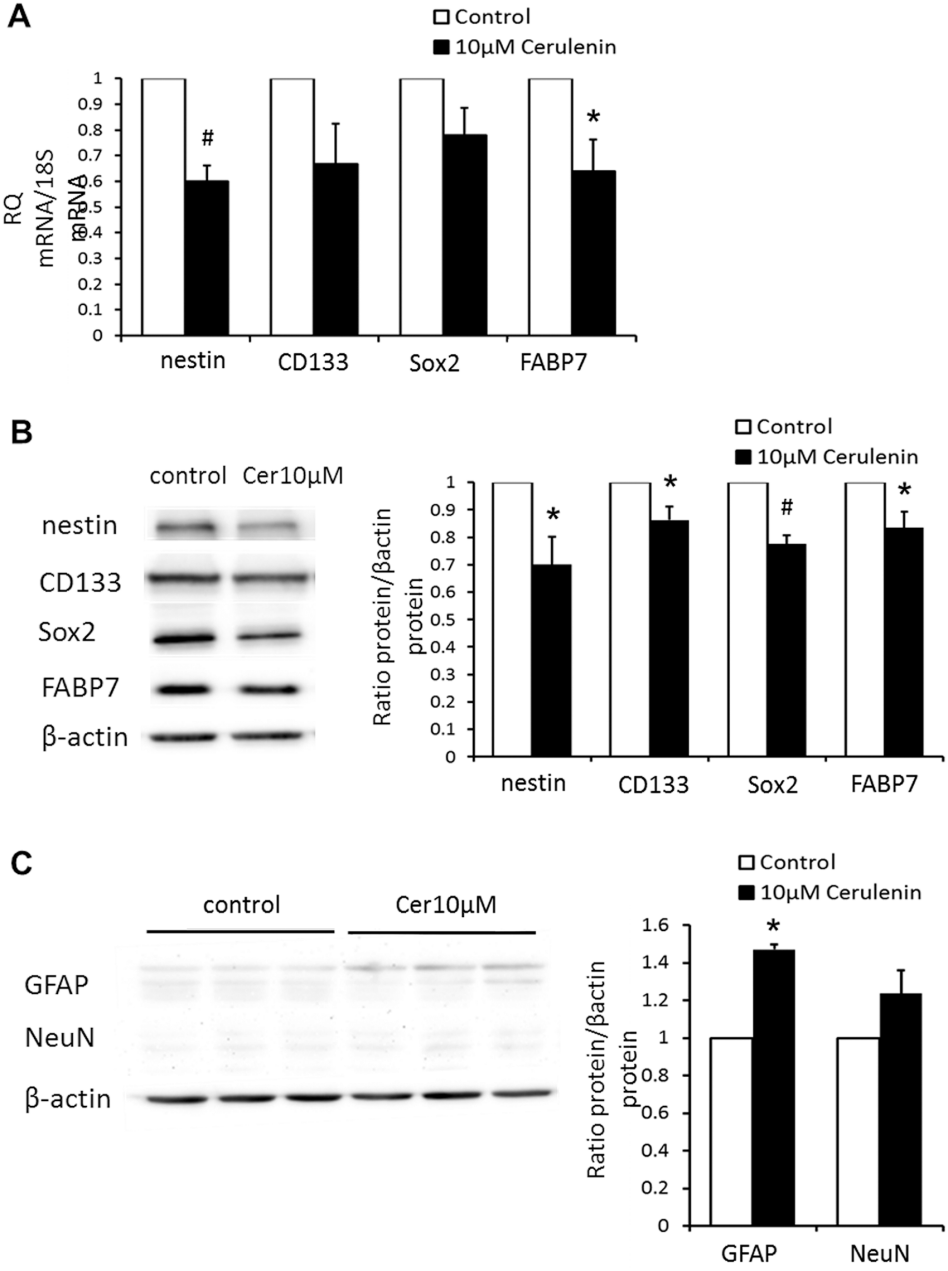
Effects of cerulenin on stemness and differentiation status. (A) qPCR results showing the expression of nestin, CD133, Sox2 and FABP7 in G144 GSC lines before and after administration of cerulenin. * P < 0.05, ^#^ P < 0.01 (B) The protein level of nestin, CD133, Sox2 and FABP7 was down-regulated by cerulenin. * P < 0.05, ^#^ P < 0.01 C. Expression of GFAP and NeuN in GSC lines before and after administration of cerulenin. * P < 0.01.

To determine whether FASN expression is associated with maintenance of the stemness of GSCs, we examined the expression of different stem cell markers in GSCs before and after cerulenin treatment. In qPCR, genes encoding nestin, CD133, and FABP7 was lower in GSCs incubated with 10 μM cerulenin for 9 d than in the control (P < 0.01 for nestin, P < 0.05 for FABP7; Fig 5A). The decrease in the expression for GSC stemness markers was further confirmed at the protein level by western blotting (P < 0.01 for Sox2, P < 0.05 for nestin, CD133, FABP7; Fig 5B). In contrast, expression of the glial differentiation marker, glial fibrillary acidic protein (GFAP), was significantly higher in GSCs after treatment with 10 μM cerulenin than that in the control (P < 0.01; Fig 5C), while the neuron-specific nuclear protein (NeuN) was unchanged in their expression (Fig 5C). Taken together, these results show that *de novo* lipogenesis controlled by FASN is essential for maintenance of GSC stemness.

## Discussion

In this study, we revealed that *de novo* lipogenesis is higher in GSCs than in differentiated non-GSCs, and that FASN is overexpressed in patient-derived GSCs. We also found that FASN expression was decreased by serum-induced differentiation, and that GSCs were more sensitive to FASN inhibition than glioblastoma cell lines. Finally, we showed that FASN inhibition reduced the invasiveness of GSCs, and expression of stemness markers, whereas it enhanced the expression of differentiation markers in GSCs. In conclusion, *de novo* fatty acid synthesis is more active in GSCs than in differentiated non-GSCs, and it is essential for maintaining of the stemness of GSCs.

Cancer cells often share characteristic metabolic abnormalities. A well-studied metabolic alteration in cancer cells is the Warburg effect increased utilization of glucose via glycolysis [31]. Another common metabolic alteration in cancer is increased glutamine metabolism. Mouse mammary tumor stem cells overexpress glutamate-cysteine ligase and glutathione synthetase, enzymes involved in the synthesis of the principal cellular antioxidant glutathione [32]. Lipids are essential for energy storage, generation of signaling molecules, and as building blocks of cellular membrane structures. Despite the growing body of evidence on alteration of glucose and glutamine metabolism being hallmarks of cancers, the role of the lipid metabolism in cancer biology is not fully understood. In this study, we determined whether *de novo* lipid synthesis is associated with the interconversion of GSCs and differentiated non-GSCs, and found that *de novo* lipogenesis was markedly higher in GSCs that in differentiated non-GSCs. In ovarian cancer spheroid cells with stem cell-like properties, acetate was utilized for *de novo* fatty acid synthesis instead of for complete oxidation [33]. Moreover, colorectal cancer stem cells can be distinguished from differentiated tumor or nomal epithelial cells because they have more lipid droplets than the other two cells [34]. Our present data suggest that *de novo* lipogenesis and cellular lipid metabolism are involved in glioma biology.

FASN expression has recently been shown to a correlate with WHO tumor grade in human glioma specimens [35]. This study reveals for the first time that FASN is overexpressed in several patient-derived GSCs. The high invasiveness of glioblastoma and poor prognosis of the patients can be attributed to the fact that glioblastomas (or high-grade gliomas) contain more cells capable of generating self-renewable spheres than low-grade gliomas [25, 30]. Of note, pharmacological inhibition of FASN by cerulenin induces death of human breast cancer cells (ZR-75-1, SKBR3, MCF-7 cells) [36], delays progression in xenograft model of ovarian cancer [37], suppresses DNA replication, and induces apoptosis in HCT116 colon carcinoma cell lines, HL60 promyelocytic leukemia cells and MCF-7 breast cancer cell line [38]. Consistent with these findings, FASN inhibition in this study markedly decreased the proliferation and migration of GSCs. The mechanism through which FASN-mediated cellular fatty acid homeostasis regulates the biological features of GSCs remains unknown.

FASN synthesizes long-chain fatty acids, mainly palmitate [29], and has a major role in the synthesis of phospholipids required for newly synthesized cellular membranes in highly proliferating tumor cells [6]. In this context, it should be noted that FABP7, which is highly expressed in malignant glioma [39] and GSCs [8], showed similar expression pattern with that of FASN during the process of GSC differentiation as shown in the present study (Fig 2B). FABP7 is highly expressed at the site of infiltration and surrounding vessels in high grade astrocytoma and down-regulation of its expression in GSCs by small interfering RNAs significantly reduces cell proliferation and migration [8]. It is still unknown whether FASN and FABP7 are cooperatively involved in the regulation of GSC proliferation, however we have recently revealed that FABP7 controls function of detergent-resistant membrane microdomains, called lipid raft, in astrocytes [40]. Interestingly, FASN is also reported to drive the production of phospholipids, mainly consisting of phosphatidylcholine and phosphatidylethanolamine, partitioning into lipid raft in prostate cancer cells [41]. Therefore it is possible that FASN and FABP7 functionally cooperate on the regulation of lipid raft mediated signaling in GSC proliferation. The possible relationship between FABP7 and FASN in lipid raft function and GSC biology should be investigated in further studies.

Our findings highlight the significance of *de novo* fatty acid synthesis in GSCs; however, the molecular mechanism underlying this process remains unclear. Further studies on the function of FASN in GSCs may provide insights into the contribution of fatty acid metabolism to the biology of glioblastoma. Identification of functional lipids and/or targeting lipid metabolism to prevent dedifferentiation or promote differentiation may lead to the development of new treatment strategies for glioblastoma.

## Supporting Information

S1 Fig Downregulation of FASN mRNA expression upon differentiation. qPCR results showing strong expression of FASN in G144, Y10 G179, Y02 and Y04 GSC lines. Upon differentiation in the presence of FBS, FASN mRNA expression, similar to that of FABP7, Sox2, and CD133 was down-regulated. * P < 0.05, ** P< 0.001 compared with control.
(TIF)

S2 Fig Characteristics of the GSC lines. Localization of FASN in mouse brain transplanted with G144 GSCs. Right, magnified image of the area was enclosed by the rectangle. Black bar = 1 mm, white bar = 50 μm.
(TIF)

S3 Fig Effects of cerulenin on glioblastoma cell viability. Cell viability was assessed 0, 24, 48 h treatment with 0, 20, 30, 40 μM cerulenin by MTS assay. U373 and U87 cell viability was significantly reduced by 40 μM cerulenin. ^#^ P < 0.01, ** P< 0.001.
(TIF)
