# Supplementary figures and images for: Inhibition of Fatty Acid Synthase Decreases Expression of Stemness Markers in Glioma Stem Cells
Source: PLoS One. 2016 Jan 25;11(1):e0147717. doi: 10.1371/journal.pone.0147717 (PMC4726602; doi:10.1371/journal.pone.0147717)

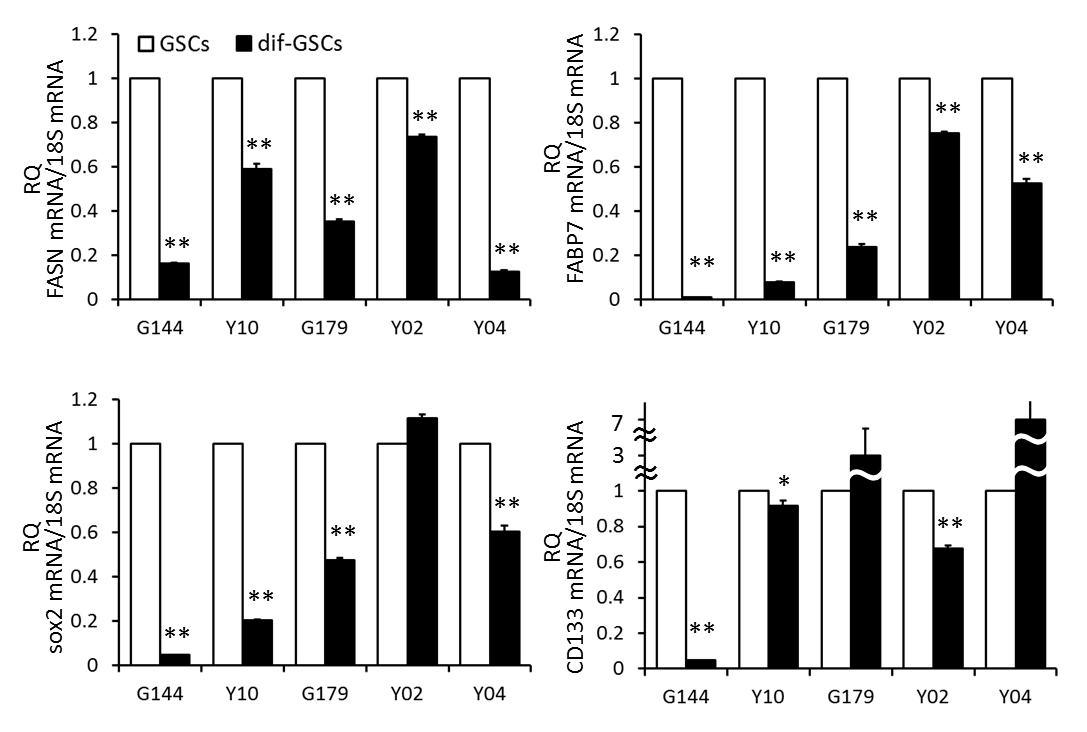

Supplement: S1 Fig — qPCR results showing strong expression of FASN in G144, Y10 G179, Y02 and Y04 GSC lines. Upon differentiation in the presence of FBS, FASN mRNA expression, similar to that of FABP7, Sox2, and CD133 was down-regulated. * P < 0.05, ** P< 0.001 compared with control. (TIF) [file pone.0147717.s001.tif]

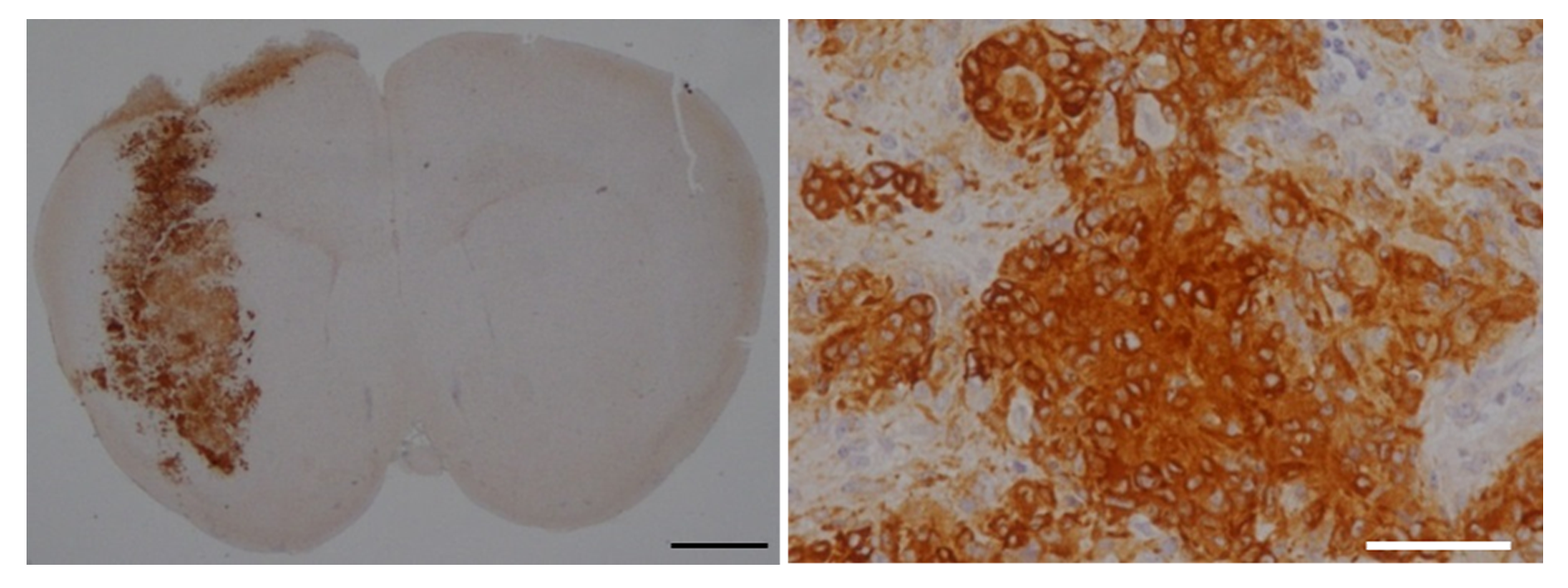

Supplement: S2 Fig — Localization of FASN in mouse brain transplanted with G144 GSCs. Right, magnified image of the area was enclosed by the rectangle. Black bar = 1 mm, white bar = 50 μm. (TIF) [file pone.0147717.s002.tif]

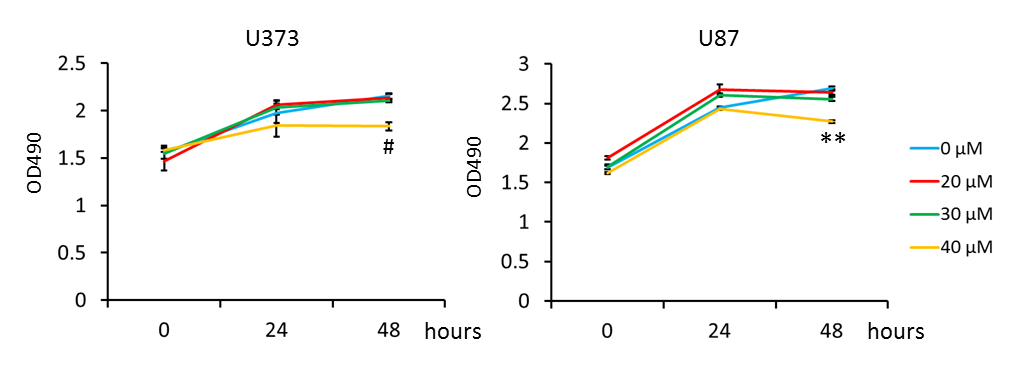

Supplement: S3 Fig — Cell viability was assessed 0, 24, 48 h treatment with 0, 20, 30, 40 μM cerulenin by MTS assay. U373 and U87 cell viability was significantly reduced by 40 μM cerulenin. # P < 0.01, ** P< 0.001. (TIF) [file pone.0147717.s003.tif]
